# Nocturnal foraging lifts time constraints in winter for migratory geese but hardly speeds up fueling

**DOI:** 10.1093/beheco/araa152

**Published:** 2021-03-25

**Authors:** Thomas K Lameris, Adriaan M Dokter, Henk P van der Jeugd, Willem Bouten, Jasper Koster, Stefan H H Sand, Coen Westerduin, Bart A Nolet

**Affiliations:** 1 Department of Animal Ecology, Netherlands Institute of Ecology (NIOO-KNAW), Droevendaalsesteeg 10, 6708 PB Wageningen, the Netherlands; 2 Theoretical and Computational Ecology, Institute for Biodiversity and Ecosystem Dynamics, University of Amsterdam, Sciencepark 904, 1098 XH Amsterdam, the Netherlands; 3 NIOZ Royal Netherlands Institute for Sea Research, Department of Coastal Systems, Den Burg, Landsdiep 4, 1797 SZ ‘t Horntje (Texel), The Netherlands; 4 Cornell Lab of Ornithology, Cornell University, Ithaca, NY, USA; 5 Vogeltrekstation—Dutch Centre for Avian Migration and Demography (NIOO-KNAW), Droevendaalsesteeg 10, 6708 PB Wageningen, the Netherlands

**Keywords:** arctic migratory birds, *Branta leucopsis*, GPS logger, hyperphagia, premigratory fueling

## Abstract

Climate warming advances the optimal timing of breeding for many animals. For migrants to start breeding earlier, a concurrent advancement of migration is required, including premigratory fueling of energy reserves. We investigate whether barnacle geese are time constrained during premigratory fueling and whether there is potential to advance or shorten the fueling period to allow an earlier migratory departure. We equipped barnacle geese with GPS trackers and accelerometers to remotely record birds’ behavior, from which we calculated time budgets. We examined how time spent foraging was affected by the available time (during daylight and moonlit nights) and thermoregulation costs. We used an energetic model to assess onset and rates of fueling and whether geese can further advance fueling by extending foraging time. We show that, during winter, when facing higher thermoregulation costs, geese consistently foraged at night, especially during moonlit nights, in order to balance their energy budgets. In spring, birds made use of the increasing day length and gained body stores by foraging longer during the day, but birds stopped foraging extensively during the night. Our model indicates that, by continuing nighttime foraging throughout spring, geese may have some leeway to advance and increase fueling rate, potentially reaching departure body mass 4 days earlier. In light of rapid climatic changes on the breeding grounds, whether this advancement can be realized and whether it will be sufficient to prevent phenological mismatches remains to be determined.

## Teaser

While geese are considered diurnal animals, accelerometers reveal that geese graze during moonlit nights in winter to maintain body weight. With the lengthening of days in spring, geese are able to fuel body stores by grazing only during the day and forego nighttime grazing. We use a model to show how much faster the geese might be able to deposit fuel stores needed for migration if they would also graze at night in spring.

## INTRODUCTION

Animals in seasonal environments can optimize reproductive success by matching reproduction timing with seasonal peaks in food availability (van Noordwijk et al. 1995). As peaks in food availability typically occur early in spring, and earlier-born offspring have a longer period to grow, early reproducing individuals often experience the highest reproductive success (Lack 1968; Sedinger and Flint 1991). Adults themselves need to deposit energy before they can lay and incubate their eggs and may actually benefit from breeding later as they cannot achieve rapid body mass gain before the peak in food availability (Prop and de Vries 1993). This is also exemplified by food supply for the female being one of the proximate drivers of laying date, with higher food availability allowing animals to reproduce earlier (Meijer and Drent 1999; Drent 2006).

Some migratory animals escape this problem by making use of multiple food peaks along their flyway as they migrate along a gradient of delayed onset of spring (a “green wave”; Drent et al. 1978; Shariatinajafabadi et al. 2014; Wang et al. 2019). This allows adults to prepare for reproduction during migration using subsequent peaks in food quality in the wintering and staging grounds, while their offspring benefits from a peak in food availability on the breeding grounds (van der Graaf et al. 2006). Under current climatic conditions, the onset of spring is advancing and, with that, the timing of peak food availability advances on the breeding grounds (Tulp and Schekkerman 2008; van Asch et al. 2012; Doiron et al. 2014; Lameris et al. 2017). To ensure overlap between chick hatching date and peak food availability, birds should advance their timing of reproduction (Visser et al. 2012). Mismatches between the timing of hatching and peak food availability have been found to have far-reaching consequences for offspring growth, survival, and eventual fitness (Visser et al. 2004; Both et al. 2006; Doiron et al. 2015; van Gils et al. 2016; Lameris et al. 2018). To advance laying dates, migrants should advance arrival on the breeding grounds (Both and Visser 2001; Lameris et al. 2018) and, as spring migration is already relatively fast, the most effective way is to advance departure from the wintering grounds (Ouwehand and Both 2017; Schmaljohann and Both 2017). For long-distance migrants, an advancement of departure is only possible when animals are in a condition to migrate and, therefore, may require an advancement of energy deposition for migration and reproduction (Lameris et al. 2017; Rakhimberdiev et al. 2018).

An animal can fuel its energy reserves by making energy intake exceed its daily energy expenditure so that the animal realizes a strongly positive energy budget, also known as hyperphagia (Bairlein and Gwinner 1994; Lindström 2003). The onset and rate of fueling can be constrained by high thermoregulatory costs and concomitant increased competition for food (e.g., in cold winter conditions; Krams et al. 2010), time available for foraging (e.g., when foraging is only possible during part of the day; Kvist and Lindström 2003, or when animals suffer from predation danger or disturbance; Bednekoff and Houston 1994; Nolet et al. 2016), digestive capacity and food quality (Prop and Vulink 1992; McWilliams et al. 1999), and food availability (Rakhimberdiev et al. 2018). In winter, when energy deposition is constrained by cold conditions, short day length, and low food quality, animals may have to forage at maximum intensity to balance energy intake with expenditure. In such stringent periods, diurnal animals, such as geese may be forced to continue foraging during nights when moonlight improves visibility (Mooij 1992; Riddington et al. 1996). During the course of spring, these constraints are reduced as food quality and quantity increases during spring (Piersma et al. 2005; Lameris et al. 2017), longer days increase time available for foraging (Prop and Vulink 1992), and higher temperatures reduce the energetic costs for thermoregulation (King and Farner 1961). Birds can then initiate fueling by keeping up maximum foraging intensity or by increasing foraging intensity in phase with a certain cue (Bairlein and Gwinner 1994), such as increasing photoperiod or temperature. When the need comes to advance departure for migration under a warming climate, animals can, therefore, only advance premigratory fueling if they have not yet reached the ceiling of maximum energy intake in early spring (Herbers 1981; Owen et al. 1992; Jeschke and Tollrian 2005).

In this paper, we study whether barnacle geese *Branta leucopsis* are time constrained during foraging and energy deposition in winter and early spring and whether they have leeway to advance the onset and increase the rate of premigratory fueling. While modeling and empirical work suggests that Arctic barnacle geese would benefit greatly from advanced timing of migration and energy deposition under Arctic climate warming, they have not advanced their date of departure from their wintering grounds yet (Lameris et al. 2017; Lameris et al. 2018). Strong variation between years in the onset of Arctic spring (Lameris et al. 2019) may currently restrain these geese to depart on migration and initiate energy deposition at the earliest opportunity.

Here, we assess potential time constraints and leeway in energy deposition by investigating how geese tune foraging time to environmental conditions. We analyze time budgets measured remotely by triaxial accelerometers in free-living barnacle geese during spring staging prior to migration. We ask 1) how the foraging duration of geese is affected by the time available for foraging (daylight and nights with ample moonlight) and thermoregulatory costs, 2) whether geese are constrained by available time for foraging to balance energy budgets in winter, and 3) how foraging duration affects the onset and rate of premigratory fueling and, hence, whether there exists potential leeway in advancing fueling.

## Methods

We collected GPS tracks with accelerometer data from 23 free-living barnacle geese, from which we derived behavior and calculated daily time budgets and foraging duration during late winter and spring. With weather data from goose-specific locations, we modeled daily thermoregulation costs, and we extracted the timing of sunset/sunrise and moon cycles to calculate the available time for foraging. Using these data, we analyzed 1) how foraging duration is affected by environmental conditions. Furthermore, we calculated total energetic costs from time budgets, which we integrated with modeled energetic intake (calculated from foraging time and metabolizable energy intake measured in the field) such that body mass trajectories could be calculated for 16 individual geese for which continuous measurements were available for the entire study period. We constructed a second and third set of body mass trajectories to simulate body mass increase under minimal and maximal foraging options, either restricted to daytime or occurring during day and night throughout spring. We used the comparison in body mass trajectories to explore 2) whether geese are time constrained to balance energy budgets in winter and 3) whether there is potential for an advanced onset and increased rate of premigratory fueling. These methods are explained in detail below, with additional methods in the Supplementary Appendices S1 and S2.

### Study population

Barnacle geese are Arctic-breeding migratory birds. In May, the population breeding in the Barents Sea region migrates northward from wintering grounds along the North and Wadden Sea coast using staging grounds in the Baltic Sea, the White Sea, Kanin peninsula, and the Barents Sea (Eichhorn et al. 2006). Foraging habitat in the wintering area (the Netherlands and Germany) consists of both manmade, agricultural pastures and natural habitats, mostly higher salt marshes as well as fresh-water marshes (Stahl et al. 2002; van der Graaf et al. 2006; Pot et al. 2019). In early spring, geese start to fuel energy reserves for migration, departing on spring migration in mid-May (Eichhorn et al. 2009). They then initiate nesting on their Arctic breeding grounds shortly after snowmelt in early June (Drent et al. 2007).

### Attachment of GPS trackers

In the colony at the Kolokolkova Bay, northern Russia (68°35’N, 52°20’E; van der Jeugd et al. 2003), we captured 40 adult female barnacle geese on the nest during the incubation period from late June to early July 2014. At capture, we measured birds’ body mass and biometrics (wing, tarsus, and head length), applied engraved colored plastic leg rings, and fitted the birds with 19-g UvA-BiTS GPS trackers (Bouten et al. 2013). We attached the GPS trackers with a 16-g Teflon harness (Lameris et al. 2017). The 35-g tracker harness combination equaled 2.3% of the average goose body mass (1495 ± 121 g). Compared to some other studies using harness attachments (Lameris and Kleyheeg 2017), we found only minimal effects on survival, migration timing, and reproductive success of tagged birds (Lameris et al. 2018). GPS trackers were programmed to collect GPS positions at an interval of 15 to 30 min in the wintering region. Following each GPS fix, a burst of 10 triaxial acceleration measurements was collected at 20 Hz (i.e., total burst length was 0.5 s). We acquired data for the period January–May 2015 from 23 individual geese, downloading data from GPS trackers using Zigbee wireless antennas and a base station, which needed to be within a range of c. 500 m from the GPS tracker (Bouten et al. 2013). We used GPS and accelerometer data for the study period, which was set from January 1, 2015, up to the onset of migratory flight (passage of the 10°E meridian, the last position in the Wadden Sea area), for most birds in mid-May 2015. The catching and tagging of free-living barnacle geese were approved by Animal Welfare Committee, protocol NIOO 14.07.

### Classification of behaviors and time budgets

From triaxial accelerometer data collected by our GPS trackers, we calculated time budgets for individual geese. A triaxial accelerometer measures movement acceleration (g-force) with respect to the earth’s gravitational field in three directions: surge (*x*), sway (*y*), and heave (*z*), and each behavioral class has its own characteristic acceleration pattern (Shamoun-Baranes et al. 2012). Using the procedure described in Shamoun-Baranes et al. (2016), we calibrated a random forest classifier (RFC) to classify foraging, flying, and other active (walking, preening, etc.) and inactive behaviors from accelerometer data. Training and validation of the RFC were conducted with a data set of accelerometer data of filmed behaviors from eight barnacle geese kept in captivity at our research facilities in Wageningen, the Netherlands, in April 2014. In this data set, segments of accelerometer data were classified into the behavioral classes inactive, active, and foraging. When a goose was sitting or standing still for a period longer than 1 s, we annotated inactive behavior. When a goose was walking (head up, not faster than 5 km/h, for longer than 1 s), we annotated active behavior. When a goose was grazing, with its head down and biting off grass tillers, for a period longer than 1 s, we annotated foraging behavior. In all other cases (e.g., other behaviors or transition between behaviors), we did not annotate the data. We annotated the behavioral class of flying for accelerometer data collected during spring migratory flights of free-living geese, for which we annotated “flying” when a goose was moving faster than 20 km/h (Shamoun-Baranes et al. 2016). Segments of annotated behavior were further segmented into samples of 10 accelerometer measurements at 20 Hz (i.e., 0.5 s). This resulted in a data set of 945 samples, including 796 inactive samples, 57 active, 44 foraging, and 48 flying. The calibration of accelerometer tags on captive geese was approved by Animal Welfare Committee, protocol NIOO 14.01.

To train our RFC, we randomly split the data set of annotated behaviors for training (40%) and testing (60%). We selected accelerometer features for the RFC by comparing the accuracy of a “pruned tree” classifier (Quinlan 1993; Witten et al. 2016) (with a confidence threshold of 0.25) for different combinations of features. Features retained in the final RFC were overall dynamic body acceleration, mean pitch (angle of the body along the *z* axis), and mean absolute time derivative of the acceleration of the *x* and *y* axes. We then trained an RFC with 50 trees with the selected features. The final RFC correctly classified 0.99 of all behaviors (*N* = 567), with 0.86 for foraging, 0.89 for active behavior, 0.99 for inactive behavior, and 1.00 for flying behavior. This resulted in a Kappa statistic (a statistical measure to compare agreement between different annotations; Sim and Wright 2005) of 0.95. We compared the performance of this RFC with an RFC using sample durations of 20 measurements (i.e., 1 s), which turned out to have a similar accuracy (mean precision of 0.91 for inactive, active, and foraging behaviors together). We then used the RFC with sample durations of 10 measurements to annotate all accelerometer data associated with every GPS fix in our data set.

**Figure 1:**
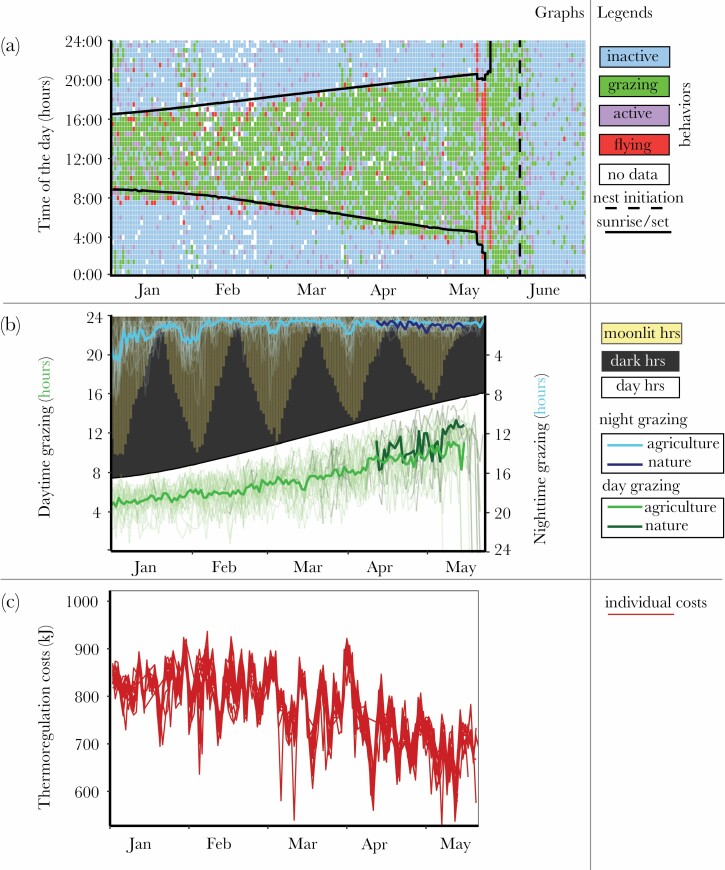
(a) Daily time budgets during the study period show time periods (of 30 min each) when geese were foraging (green), resting (blue), flying (red), or active (purple) or when no data was available (white), shown for one individual bird (bird 6087) as example. Black lines delineate the time of sunrise (lower) and sunset (upper); black dashed lines show the day of nest initiation. The bird departs on migration from the wintering grounds in mid-May, after which it flies to the Arctic where it encounters 24 h of daylight. (b) Foraging duration in hours during the study period by individual birds (thin lines) and the population average (thick lines) during daytime (green, left *y* axis) and nighttime (blue, right *y* axis). Foraging duration is differentiated between agricultural pastures (light green/light blue) and natural habitats (dark green/dark blue). The white area shows daytime hours (left *y* axis), the black shadowed area shows nighttime hours in which yellow bars show the hours during nighttime, during which the moon was visible (right *y* axis). (c) Daily thermoregulation costs for individual birds (thin red lines) during spring.

To make data sets with different intervals between GPS fixes comparable, we resampled all data to include only one GPS fix every 30 min. We considered the sampled behavior to be representative for the time interval between one and the following GPS fix. From these data, we generated daily time budgets for every individual bird (Figure 1a).

### Day length, weather, and land use data

For every day in 2015, we collected data on sunrise and sunset for the locations of individual geese using the National Oceanic and Atmospheric Administration (NOAA) sunrise and sunset calculator tool (Cornwall et al. 2014) via the function “sunrise.set” in the R package “StreamMetabolism” (Sefick Jr 2009). From these data, we calculated day length (time between sunrise and sunset) and determined whether GPS fixes were taken during daytime or during nighttime. We collected data on moonrise, moonset, and fraction of moon visible for every day using the average location for the population (53°52’N, 7°18’E) in the United States Naval Observatory (USNO) moonrise and moonset calculator (Naval Meteorology and Oceanography Command 2011). From sunrise, sunset, moonrise, and moonset data, the duration per night during which the moon was visible was calculated. As the fraction of moon visible correlated strongly with the duration per night during which the moon was visible, we subsequently only used the duration per night during which the moon was visible. Data on daily average temperature, cloud cover, U (zonal velocity, i.e., the component of the horizontal wind toward the east), and V (meridional velocity, i.e., the component of the horizontal wind toward the north) wind components at 10 m and long- and short-wave radiation were collected at 6-h intervals for locations of individual geese from the National Centers for Environmental Prediction (NCEP) reanalysis numerical weather model (Kalnay et al. 1996) using the R package “RNCEP” (Kemp et al. 2012). For every location where a bird was foraging, we categorized locations by habitat, using the CORINE data set from 2006 (Büttner et al. 2004; Büttner and Kosztra 2007), and distinguished between agricultural area (arable land, pastures, and heterogeneous agricultural areas) and natural areas (natural grassland, inland marshes, and salt marshes). For 6.8% (149.445) of the fixes where a bird was classified as foraging, we encountered other classes of land use. Using Google Earth, we determined whether this was due to an erroneous land cover classification (6.5%), after which we corrected this by hand, or whether the foraging behavior was classified incorrectly (0.3%). The latter was often the case when birds were sitting on the open sea as waves can result in an accelerometer signal similar to foraging and, for these points, we corrected the behavior to resting (Shamoun-Baranes et al. 2011, 2016).

### Body mass data from captured birds

For the comparison of modeled body mass from body mass trajectories (see below), we compiled data on body mass taken from 484 adult female geese, which were captured between January and April in the Netherlands and Germany during 1979, 1989, and 2000–2017 (see Supplementary Appendix S2).

### Modeling costs of thermoregulation and activity

In order to infer energetic expenditure, we modeled thermoregulation and activity costs by combining time budgets derived from accelerometer data and local weather data in a bioenergetics model. While these models come with some uncertainties and may, therefore, not be accurate enough to make comparisons between individual birds, they do reflect observed patterns in body mass trajectories (Dokter et al. 2018). For one GPS fix (*i*) every 30 min, we calculated the energy needed for existence (energy costs depending on activities; *E*_*e*_), thermoregulation energy costs (*E*_*t*_), and the total energy costs (*E*_s_). We calculated energy costs for a 1600-g barnacle goose for the entire study period.

Thermoregulation costs *E*_*t*_ are the costs to maintain a relatively constant body temperature when environmental temperature drops below the thermoneutral zone, for which the lower critical temperature for barnacle geese lies close to 10 °C (Eichhorn G et al. unpublished data). We followed the model developed by Cartar and Morrison (1997), which calculates the thermoregulation costs *E*_*t*_ as a function of body mass, ambient temperature, wind speed at 10 m, and global radiation at the time and location of fix *i*. Our parameterization was similar to Baveco et al. (2011), but we used the plumage resistance measured specifically for barnacle geese (van der Graaf et al. 2001) and a bird height of 0.15 m.

We calculated existence energy *E*_*e*_ for every GPS fix *i* as

$$\begin{array}{l} E_{e}=BMR\;\times \;b\;\times \;t \end{array}$$ (1)

Where BMR is the basal metabolic rate of an individual bird, *b* the behavior-specific multiplier (inactive = 1.5, active = 1.9, foraging = 1.6; Stahl 2001) for behaviors determined using accelerometer data (see above) and *t* is the time interval from the GPS fix *i* until the following GPS fix *i* + 1. For flying behavior determined from accelerometer data, we set energetic costs at 0, as we calculated flight costs separately (see below).

BMR varies proportionally with an individual’s body mass (Daan et al. 1989) following:

$$\begin{array}{l} BMR_{i}=(W_{i}\;/\;W_{ref})\;\times \;BMR_{ref} \end{array}$$ (2)

with BMR_ref_ = 5.536 W, *W*_*i*_ the body mass at fix *i*, and *W*_ref_ = 1825 g (Nolet et al. 1992). To calculate thermoregulation costs, we used the BMR for our set body mass (4.853 W for a 1600-g barnacle goose).

As GPS-fix intervals of 30 min cannot detect short periods of flight, we calculated whether a bird had flown a certain distance based on a minimum ground speed. When it was likely that a goose had flown this distance, we calculated the energetic expenditure based on airspeed, taking into account wind conditions (see Supplementary Appendix S1 for full methods).

We assumed that heat generated during activities can be used for thermoregulation (Paladino and King 1984) and, only when *E*_*t*_*> E*_*e*_, the bird pays additional thermoregulation costs. Total energy costs were calculated as:


$$\begin{array}{l} E_{s}=max\left(E_{t};E_{e}\right) \end{array}$$ (3)

### Body mass trajectories

We modeled body mass trajectories (BMTs) for the 16 individual geese for which continuous GPS and accelerometer data were available for the entire study period. We modeled BMTs from energy budgets following Dokter et al. (2018). Baseline BMTs were modeled from 1) time budgets from GPS trackers, 2) metabolizable energy intake from field data, and 3) thermoregulation and existence energy from weather data. For a given GPS fix (*i*), we calculated the bird’s body mass (*W*_*i*_) as a result of body mass at the previous GPS fix (*W*_*i* − 1_) plus the intake of metabolized energy (*I*_*i*_) minus the total energy costs (*E*_*s*_), multiplied by the efficiency of body mass storage or use. We calculated the rate of metabolized energy intake rate (*I*) from field data, which included the quality of forage plants, dropping rate (rate of defecation events in foraging geese; Dokter et al. 2017), and dropping mass, and how these parameters changed during spring. For calculations of activity costs, we here used the body mass-dependent BMR as outlined above, based on the bird’s body mass at the previous GPS fix (*W*_*i −* 1_). When energy is metabolized above the energetic needs, birds store the production energy (*P*_*i*_) in stores with an efficiency of 0.8; when energetic costs exceed the metabolized energy, body stores are burned to gain energy with an efficiency of 1 (Blaxter 1989). Energy and mass were converted (either for storing or burning body reserves) using the energy density of deposited tissues as measured for female pink-footed geese (29 kJ/g; Madsen and Klaassen 2006). We used a body size-specific starting body mass based on body masses measured in barnacle geese during winter (see Supplementary Material for full methods).

We calculated the rate of metabolizable energy intake *I* from field data collected in a representative wintering site of migratory barnacle geese in spring 2016 and 2017 (see Supplementary Appendix S2). To correct for differences between years, we calculated linear regressions between *I* and growing degree days (GDD; Lameris et al. 2017). As metabolizable energy intake can differ strongly between natural and agricultural foraging habitats (Dokter et al. 2018; Pot et al. 2019), regressions for both habitats were determined separately (Supplementary Figure S1). From these regressions, we predicted habitat-specific potential metabolizable energy intake rate (PI) for tagged geese foraging in either of these habitats based on local GDD. As dropping rates were observed for geese that were displaying a combination of foraging and active behavior rather than dropping rates of geese that were only foraging (see Supplementary Appendix S2), we multiplied *PI* with foraging time plus the time active on habitats suitable for foraging (agricultural pastures or natural grasslands) to calculate metabolizable energy intake *I*_*i*_.

Given the flexible foraging duration during nighttime (see Results), we used BMTs to test the importance of nighttime foraging for 1) balancing energy budgets and 2) increasing the rate of body mass increase in two scenarios with changed time budgets. These included 1) a “no nighttime foraging” scenario, where geese would only be inactive during the night, and not graze, and 2) a “maximum foraging” scenario where geese would forage maximally during moonlit nights throughout the entire study period. For these scenarios, we modeled BMTs following changes in time budgets (which affect existence energy and energy intake rate). For the first scenario, we changed all behaviors during nighttime to “inactive” behavior. For the second scenario, at the level of individual geese, we used time budgets during the nights in January (the first moon cycle between January 1 and 30, during which longest nighttime foraging durations were achieved) to replace nighttime time budgets during nights in the following moon cycles. For example, time budgets of the nights of January 31 to February 1, March 2 and 3, April 1 and 2, and May 1 and 2 were replaced by the time budgets of the nights of January 1 and 2; time budgets of the nights of February 1 and 2, March 3 and 4, April 2 and 3, and May 2 and 3 were replaced by the time budgets of the nights of January 2 and 3; and so on.

### Statistics

First, we aimed to 1) test how available time for foraging and thermoregulation costs affected foraging duration both during daytime and nighttime. Second, we aimed to 2) test whether geese are constrained by available time for foraging to balance energy budgets in winter. Here, we specifically aimed to quantify the importance of nighttime foraging to balance energy budgets by assessing how body mass decline and minimum body mass was affected by time budgets under the scenario of no nighttime foraging. Third, we aimed to 3) test whether there exists potential leeway in advancing premigratory fueling. Specifically, we aimed to test when geese started the deposition of energy (measured by an increase of foraging duration and body mass), when they reached their maximum (departure) body mass, and how this was affected by time budgets under scenarios of no nighttime foraging or maximum foraging.

1) We tested drivers of foraging duration using linear mixed models using the package “nlme” in R 3.0.2 (R Core Team 2020). Candidate models were constructed from all possible combinations of predictor variables, including ecologically meaningful interactions. Models were compared using Akaike’s information criterion (AICc; Burnham and Anderson 2002). We chose the model with the lowest AICc value as our final model (Table 1), where models within 2 ΔAICc were considered as competitive but only if these did not contain additive parameters (Arnold 2010). Parameter estimates were obtained by model averaging using the package MuMln (Bartoń 2019). Support of the selected model (or models) relative to next best model was calculated from the ratio of model weights (Burnham et al. 2011). We ran separate model sets for foraging duration during daytime, nighttime, and daytime and nighttime combined. We included predictor variables habitat (agricultural or natural habitat) and day length (for daytime models), day-of-the-year and hours of moonlight (for nighttime models) or day length and hours of moonlight (for daytime and nighttime combined), and individual birds as a random intercept in all models. Variance inflation factors ranged between 1.09 (habitat) and 1.27 (day-of-the-year), which was below the threshold of 2.5, above which considerable collinearity occurs (Johnston et al. 2018). We found high collinearity between thermoregulation costs and day-of-the-year (0.65, Spearman’s rank correlation coefficient), as well as between thermoregulation costs and day length (0.66), because thermoregulation costs decreased over time. We, therefore, tested for the effect of thermoregulation costs on foraging time in an additional analysis with similar predictor variables as outlined above as well as including thermoregulation costs, but where we only included the months January and February. Over this shorter period with constant high thermoregulation costs, collinearity was much lower (0.05 with day-of-the-year and 0.06 with day length). Habitat type was not taken into account in these models, as almost all foraging occurred on agricultural pastures during these months. Model assumptions of linearity, normality, independence, and equality of variance were assessed visually using residual plots, *Q*–*Q* plots, and correlograms. We encountered models to violate assumptions of independence and equality of variance. We corrected for temporal autocorrelation setting a correlation structure with the order of observations as a covariate, using corAR1 in the package “nlme.” We checked for the best variance structure by comparing the AICc of full models (including all predictor variables) with different variance structures, including fixed variance, different variances per habitat, power, and constant power or exponential of the variance covariate (day length of day-of-the-year). The best variance structures (in full models with lowest AICc) were power of day length with different variances per habitat for daytime models and daytime and nighttime combined and power of day-of-the-year for night time models.
2) We determined the moment at which the minimum body mass was reached in the “baseline” and the “no nighttime foraging” scenarios and tested the difference in minimum body mass between these two scenarios using a Wilcoxon signed-rank test, for which we report the *V* statistic. We tested the difference in body mass decrease between scenarios in linear mixed models using the output body mass from the BMTs up to the day at which the minimum body mass was reached in the baseline BMT (as there was no significant difference in the date at which the minimum body mass was reached between scenarios, pairwise Wilcoxon signed-rank test *V* = 40, degrees of freedom [df] = 14, *P* = 0.20). We constructed candidate models, including day-of-the-year, and the interaction between day-of-the-year and scenario as predictor variables and individual birds as random intercept. We did not include scenario as predictor variable as both BMT scenarios used the same starting body masses and, therefore, the intercept in a linear regression of body mass should not differ between scenarios. We conducted model selection as outlined above.
3) To pinpoint the onset of premigratory fueling, we tested at which day the total foraging time and BMTs started to increase by determining temporal breaking points for every individual bird in segmented linear regression models (Bacon and Watts 1971), including day-of-the-year as a segmented fixed effect. We selected the breaking point for day-of-the-year, which led to the maximum likelihood estimates for all parameters in the model using the “optimize” function in R, with a starting value for the breaking point of 4. For comparison, we determined the breaking point for the complete data set of body masses from wild birds in a similar fashion. In the baseline BMTs, we determined the maximum body mass as “departure body mass” and the moment at which this was reached. In all cases, this body mass was reached on the day of departure. We then determined when the individual “maximum foraging BMTs” reached these same departure body masses. Finally, we calculated the time between the breaking point (onset of premigratory fueling) and the time at which departure body mass was reached at the individual level as the “energy deposition period.” We conducted pairwise Wilcoxon signed-rank tests to compare the onset of premigratory fueling, moment of departure body mass, and time required for energy deposition between the baseline BMTs and maximum foraging BMTs.

**Table 1:** Linear mixed effect models for daytime foraging (DG), nighttime foraging (NG), and total foraging duration (TG)

	Model	df	AICc	Delta AICc	Model weight
	(A) Daytime foraging duration (DG)				
1	**DG ~ DL + H + DL** × **H**	9	33 894.1	0	0.975
2	DG ~ DL + H	8	33 901.5	7.33	0.025
3	DG ~ DL	7	33 914.5	20.33	0
4	DG ~ H	7	34 625.3	731.11	0
5	DG ~ 1	6	34 633.3	739.11	0
	(B) Nighttime foraging duration (NG)				
1	**NG ~ DY + H + MT + MT** × **H**	9	31 236.6	0	0.593
2	NG ~ DY + H + MT	8	31 237.5	0.89	0.38
3	NG ~ DY + MT	7	31 244.3	7.73	0.012
4	NG ~ H + MT + MT × H	8	31 245.0	8.35	0.009
5	NG ~ H + MT	7	31 246.1	9.52	0.005
6	NG ~ MT	6	31 250.6	14.02	0.001
7	NG ~ H + DY	7	31 296.7	60.11	0
8	NG ~ DY	6	31 302.9	66.30	0
9	NG ~ H	6	31 330.7	94.09	0
10	NG ~ 1	5	31 333.7	97.13	0
	(C) Total foraging duration (TG)				
1	**TG ~ DL + H + MT + MT** × **H + DL** × **H**	11	36 004.7	0	0.932
2	TG ~ H + MT + MT × H	10	36 010.3	5.54	0.058
3	TG ~ H + MT + DL + DL × H	10	36 014.0	9.27	0.009
4	TG ~ DL + H + MT + MT × H	9	36 022.1	17.32	0
5	TG ~ DL + MT	8	36 036.5	31.80	0
6	TG ~ DL + H + DL × H	9	36 040.3	35.54	0
7	TG ~ DL + H	8	36 047.8	43.02	0
8	TG ~ DL	7	36 061.2	56.44	0
9	TG ~ H + MT + MT × H	9	36 420.0	415.24	0
10	TG ~ H + MT	8	36 426.6	421.81	0
11	TG ~ H	7	36 428.1	423.32	0
12	TG ~ MT	7	36 435.4	430.63	0
13	TG ~ 1	6	36 442.2	437.43	0

Models include fixed effects day length (DL), day-of-the-year (DY), habitat type (H), number of moonlit hours (MT), including interactions between day length and habitat (DL × H) and number of moonlit hours and habitat (MT x H). Goose identity is included as random intercept. Models are ordered from lowest to highest AICc values, with the best performing model marked bold.

### Sensitivity analysis

We evaluated the sensitivity of body mass gain in BMT models (and, specifically, the timing of departure body mass) to changes in the parameter values of potential metabolizable energy intake rate (PI) and basal metabolic rate (BMR_ref_). We generated normal distributions for both parameters. For PI, this distribution was centered around the estimated PI–GGD relationship (see above), and the width of the distribution was given by the standard deviation (SD) of the intercept coefficient of this relationship (with data from agricultural and natural habitats pooled for simplicity). For BMR_ref_, this was a normal distribution based on the data from Nolet et al. (1992; average = 5.54 W, SD = 0.42, *n* = 5).

First, we ran simulations of the BMTs (both baseline and maximum foraging BMTs) either sampling parameter values only for PI (500 simulations), only for BMR_ref_ (500 simulations), or for both parameters (1000 simulations; Supplementary Figure S2). Second, we ran a set of simulations of BMTs with new parameter values calculated by either adding or subtracting 2 SDs from their intercept. For all individual birds in simulations, we calculated 1) the moment of maximum body mass in baseline BMTs, 2) the moment when individuals reached the same “departure body mass” in maximum foraging BMTs as in baseline BMTs, and 3) the time difference between these two moments. In simulations where PI was reduced by more than 0.5 SD, body mass of some individuals in baseline BMTs quickly decreased over time, and the maximum body mass was found on January 1. As this was not the case in maximum foraging BMTs, the difference in departure body mass between baseline BMTs and maximum foraging BMTs become strongly negative for such individuals. Parameters leading to these unrealistic scenarios were omitted from the analysis.

## RESULTS

### Foraging duration and available time for foraging

Geese grazed mostly during daylight hours, initiating long bouts of foraging shortly after dawn and continuing foraging until dusk, and rested long periods during the night (Figure 1a). Geese increased daytime foraging duration in agricultural habitats with increasing daylight hours (regression coefficient β = 35.3 ± 0.9 min increased foraging duration per hour of day length; Figure 1b; Tables 1 and 2).

**Table 2:** Coefficients from the most parsimonious models for foraging time during daytime and nighttime

	Estimate	SE
(I) Models for entire spring period (January–May)		
(A) Foraging duration (min) during daytime (df = 9, residuals = 2901)		
Intercept agricultural habitat	34.3	13.5
Day length (h) in agricultural habitat	35.3	0.9
Intercept natural habitat	−74.9	64.6
Day length (h) in natural habitat	45.5	4.8
(B) Foraging duration (min) during night time (df = 9, residuals = 2901)		
Intercept agricultural habitat	43.6	6.5
Day-of-the-year (day)	−0.2	0.05
Moonlit hours (h) agricultural habitat	3.6	0.4
Intercept natural habitat	56.2	7.1
Moonlit hours (h) natural habitat	2.6	1.2
(C) Total foraging duration (min; df = 11, residuals = 2952)		
Intercept agricultural habitat	40.1	24.0
Day length (h) agricultural habitat	38.0	1.5
Moonlit hours (h) agricultural habitat	5.3	0.9
Intercept natural habitat	−1.7	77.9
Day length (h) natural habitat	46.3	5.3
Moonlit hours (h) natural habitat	−2.7	3.0
(II) Models for winter months (January–February)		
(D) Foraging duration (min) during daytime (df = 7, residuals = 1262)		
Intercept	46.2	23.3
Day length (h)	33.8	2.4
(E) Foraging duration (min) during night time (df = 8, residuals = 1262)		
Intercept	−28.9	35.8
Day-of-the-year (day)	−1.7	0.2
Moonlit hours (h)	4.6	0.7
Thermoregulation costs (kJ)	0.2	0.04
(F) Total foraging duration (min; df = 9, residuals = 1262)		
Intercept	156.6	73.4
Day length (h)	4.3	4.5
Moonlit hours (h)	6.4	1.0
Thermoregulation costs (kJ)	0.2	0.1

SE, standard error.

Nighttime foraging duration in agricultural habitats occurred mostly under moonlit conditions as geese spent up to 4–6 h foraging in nights around full moon and increased their nighttime foraging duration with hours of moonlight (β = 3.6 ± 0.4 min/h of moonlight; Figure 1b; Tables 1 and 2). Geese decreased their nighttime foraging duration over the study period (β = 0.2 ± 0.05 min decrease foraging per day; Tables 1 and 2) and grazed longest during moonlit nights in January–February (Figure 1b; Tables 1 and 2). In April and May, nighttime foraging of geese occurred mostly in early morning before sunrise during civil twilight (Figure 1a)

Compared to agricultural habitats, geese in natural habitats increased their daytime foraging duration faster over the study period following increasing daylight hours (β = 45.5 ± 4.8 min increase foraging duration per hour of day length; the model with interaction term day length and habitat is 39.1 times more likely than the model without interaction term; Table 1). In natural habitats, geese foraged longer during the night as compared to agricultural habitats (β = 56.2 ± 7.1 min foraging duration per night vs. 43.6 ± 6.5 in agricultural habitats; the model with habitat term is 30.6 times more likely than the model without habitat term; Table 1) but responded less to moonlit hours (β = 2.6 ± 1.2 min increase per hour of moonlight; the model with interaction term is 1.6 times more likely than the model without interaction term; Table 1).

### Foraging duration and energetic costs

In January and February, foraging duration during daytime did not vary with thermoregulation costs (the model without thermoregulation is 19.4 times more likely than the model with thermoregulation; Supplementary Table S1). In contrast, nighttime foraging duration increased with higher thermoregulation costs (β = 0.15 ± 0.04 min increase in foraging time per kilojoule; the model with thermoregulation is 30.4 times more likely than the model without thermoregulation; Figure 1c; Table 2; Supplementary Table S1). The model for total foraging time (day and night combined) also showed foraging duration to increase with thermoregulation costs (β = 0.22 ± 0.07 min increase in foraging time per kilojoule; the model with thermoregulation is 12.0 times more likely than the model without thermoregulation; Table 2 and Supplementary Table S1).

### Nighttime foraging and winter energy budgets

Lowest body masses were reached at March 4 ± 13 days. Lowest body mass in our baseline BMTs was 1427 ± 96 g, which was significantly higher than the lowest body mass when birds would not forage at night (“no nighttime foraging” BMTs: 1316 ± 81 g, *V* = 0, df = 14, *P* < 0.001). Linear mixed models revealed that body mass decreased faster when birds would not forage at night (−6.10 ± 0.06 g/day) compared to the baseline BMTs (−3.8 ± 0.06 g/day; Supplementary Table S2).

### Onset of premigratory fueling and departure

**Figure 2:**
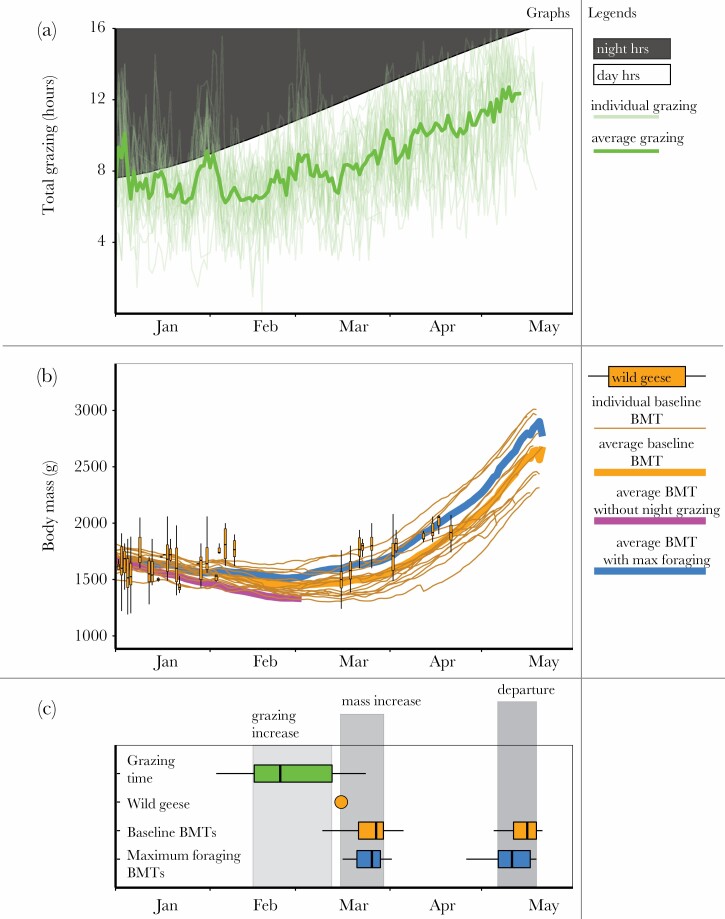
(a) Total foraging duration of barnacle geese during the study period by individual birds (thin lines) and the population average (thick light-green line). (b) Modeled baseline BMTs over the study period for individual birds (thin orange lines) and the population average (thick orange line), with vertical boxplots along the trajectories showing observed body masses from wild geese, measured at the same day of the year between 1980 and 2017. The thick blue line shows the population average for the “maximum foraging” scenario BMTs; the thick violet line shows the population average for the “no nighttime foraging” scenario BMTs. (c) Results from breaking point analyses: boxplots on the left show the distribution of break points for individual onsets of increase in foraging time (green), the onset in body mass increase measured in wild geese (orange dot), onset in body mass increase in baseline BMTs (orange) and “maximum foraging” scenario BMTs (blue). Boxplots on the right show the timing of departure of tracked geese at which they reached departure body mass (orange) and the moment at which departure body mass was reached in “maximum foraging” scenario BMTs (blue).

Barnacle geese started to increase daily foraging duration from February 28 onwards (Figure 2a). BMTs (Figure 2b) showed close to stable body mass during the period January to mid-March, with body mass starting to increase from March 25 ± 8 days. BMTs under the maximum foraging scenario showed a 2-day advancement in the onset of body mass increase at March 23 ± 8 days (*V* = 12, df = 14, *P* = 0.004; Figure 2c). From body mass measurements on wild female barnacle geese, we find an increase in body mass starting at least mid-March but possibly earlier as we lack measurements during February. Measured body masses correlated with average modeled BMT at the same day-of-the-year (Pearson correlation = 0.27, *t* = 6.0, df = 482, *P* < 0.001).

Tracked birds departed on spring migration from the Wadden Sea region on May 14 ± 7 days, with an average departure body mass of 2527 ± 337 g as modeled by the baseline BMTs. In the maximum foraging BMTs, we find that the utilization of maximum available foraging time would allow individual birds to reach these same values 4 days earlier at May 10 ± 8 days (*V* = 0, df = 14, *P* = 0.001; Figure 2c). As a result, BMTs under the maximum foraging scenario showed a shorter energy deposition period (48.0 ± 8.3 days) compared to the baseline BMTs (50.1 ± 8.2 days, *V* = 6, df = 14, *P* = 0.002).

### Sensitivity analysis

**Figure 3:**
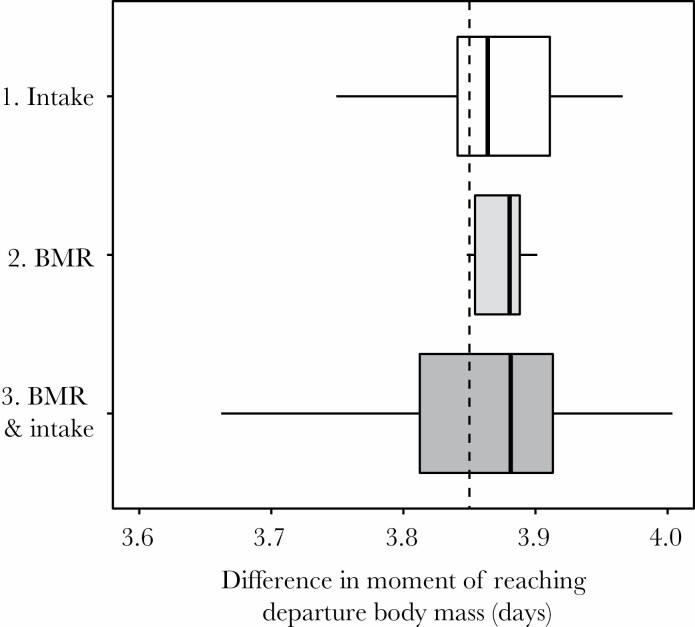
Boxplots showing the differences in reaching “departure body mass” in baseline BMTs and maximum foraging BMTs. Uncertainty in the model estimates was quantified by randomly sampling parameter values from their respective probability distributions for (1) potential intake rate PI (500 simulations), metabolizable intake rate BMR_ref_ (500 simulations), and (3) both parameters (1000 simulations). The vertical dotted line shows the difference in the date of reaching “departure body mass” between the baseline BMT and maximum foraging BMT with the original parameters.

The effect of change of parameter values for potential intake rate PI and basal metabolic rate BMR_ref_ are shown in Table 3. New parameter values did not result in large changes in the timing of maximum body mass (in baseline BMTs) and departure body mass (in maximum foraging BMTs) and the difference between those (Figure 3).

**Table 3:** Results of the sensitivity analysis, where the potential intake rate PI and basal metabolic rate BMR_ref_ were increased (+) or reduced (−) by 2 SDs

Changed parameter	Direction	Date of reaching departure body mass		Difference in departure (days)
		Baseline BMT	Maximum foraging BMT	
Baseline BMT	0	14/05 18:08	10/05 21:46	3.85
Potential intake rate PI	+	14/05 14:16	10/05 16:23	3.91
	−	17/05 19:40	13/05 21:37	3.84
Basal metabolic rate BMR_ref_	+	14/05 14:24	10/05 16:48	3.89
	−	14/05 14:08	10/05 17:11	3.87

Results show the average date of reaching “departure body mass” in baseline BMTs and maximum foraging BMTs, and the average difference in this timing between the models.

## Discussion

Using accelerometer-derived time budgets, we show that wintering barnacle geese adjusted their foraging duration to the available time for foraging during daytime. Geese supplemented foraging duration by also foraging during moonlit nights, most noticeably when facing higher energy expenditure costs. From late February onwards, geese increased daytime foraging duration with the lengthening of days and started to realize energy deposition from mid-March. We discuss how geese adjust foraging duration to balance their energy budget and what this means for the potential of geese to advance the onset of premigratory fueling in a warming climate.

### Energy budgets are balanced by adjusting foraging duration

During the day, barnacle geese actively grazed for a stable fraction (66%) of the daylight hours throughout the study period. This appears a relatively low fraction of the day as compared to observational studies, which typically find fractions between 80% and 90% (Ebbinge et al. 1975; Black et al. 1991; Owen et al. 1992). Using accelerometers, we classify short periods (of 0.5 s or longer) of standstill as resting, while, in observational studies, only prolonged periods of sitting are classified as resting. The fact that foraging takes up a stable fraction of daylight hours suggests that geese can only feed during a proportion of daylight hours. The remainder of the time may be taken up by other important activities, such as commuting to the feeding grounds, preening and bathing, social interactions, and being vigilant for predators (Metcalfe and Furness 1984). Geese may also be facing a digestive bottleneck (Sedinger and Raveling 1988; Kersten and Visser 1996), meaning that the time needed to digest the food exceeds the time required for intake. However, this is unlikely to be the case in late winter (January–February), when foraging intake and food retention times are low (Prop and Vulink 1992; Dokter et al. 2018). In either case, barnacle geese do not appear to have a large potential to increase foraging time during the day.

During winter, when short day length reduced time available for foraging during the day, geese also grazed during the night, and grazed more during moonlit nights. Nighttime foraging has been described for multiple species of shorebirds and waterfowl (Mcneill et al. 1992). For birds that forage in intertidal systems, the timing of foraging bouts is principally governed by tides (Evans 1976; van der Kolk et al. 2020) and many shorebird species are well capable of foraging in the dark (Thomas et al. 2006). Also many species of aquatic foraging waterfowl, such as ducks and swans, typically feed at night (Guillemain et al. 2002; Nolet and Klaassen 2005). For many of those species, foraging at night is considered to be preferred due to lower predation risk and, in some cases, increased food availability at night (Mcneill et al. 1992). From observational studies, it is well known that geese forage during the night in winter, especially during moonlit nights (Ebbinge et al. 1975; Mooij 1992; Riddington et al. 1996; McWilliams and Raveling 1998; Tinkler et al. 2009; Daniels et al. 2019). In contrast to nocturnal foraging in other waterbirds, it has been proposed that geese forage during the night to supplement daytime foraging (Mcneill et al. 1992), but lack of data on seasonal patterns in nocturnal foraging have warranted strong conclusions. Our data show extensive nighttime foraging during winter months but decreasing nighttime foraging over the course of spring, which supports the idea that geese need to supplement daytime foraging by foraging at night during winter (Owen 1972; Ebbinge et al. 1975; Riddington et al. 1996). In the winter months, geese grazed more during nights with higher thermoregulatory costs. During moonlit nights (>12 moonlit hours) in late winter, on average, 26% ± 19% of daily foraging duration was during nighttime, which suggests that foraging during the night can add substantially to the daily intake of geese (Tinkler et al. 2009). Our BMTs also allow us to quantify the importance of nocturnal foraging. These show that barnacle geese relied heavily upon nighttime foraging to balance energy budgets as, without nighttime foraging, body mass decline during winter was twice as rapid, and geese reached body masses more than 100 g lower than in baseline BMTs.

By foraging during daytime and nighttime, barnacle geese kept to a constant daily foraging time of around 7 h/day during late winter. As spring progressed, nighttime foraging became less prevalent, probably as day length and food quality increased, which allowed geese to reach their required daily intake during day time. We thus argue that barnacle geese are not necessarily constrained in foraging time but aim for a certain daily energy intake. This is stressed by our finding that, at least during winter, geese match foraging duration to energetic costs, which has also been shown for brent geese (Dokter et al. 2018). As such, geese appear to be able to increase their foraging duration when needed. This is also supported by differences that we find in foraging duration between agricultural and natural habitats. In the course of spring, many barnacle geese switch from foraging in agricultural habitats to natural habitats, such as salt marshes (Pot et al. 2019). Historically, this habitat switch occurred at the moment that food quality in natural habitats equaled the food quality in agricultural habitats (Prins and Ydenberg 1985). Currently, food quality in natural habitats is consistently lower than in agricultural habitats (Eichhorn et al. 2012; Pot et al. 2019), and barnacle geese respond by increasing their foraging duration in natural habitats (Pot et al. 2019).

### Constraints and flexibility in the potential for advancing premigratory fueling

We observe that geese balance their foraging duration with their energy needs and, therefore, do not appear to be strongly time constrained (Jeschke and Tollrian 2005; Dokter et al. 2018), at least not after January when foraging duration at night decreases. In spring, this could also imply that there is leeway to further increase foraging time, thereby advancing the onset of premigratory fueling, and increase the rate of energy deposition, which could facilitate an earlier migratory departure in a warming climate (Lameris et al. 2017).

To explore such a scenario, we modeled BMTs for individual geese based on accelerometer-derived time budgets, for which we use an extensive modeling exercise combining the modeling of energy intake and expenditure. We find a rather close match between body mass measurements from geese captured during energy deposition and our average baseline BMT. This allows us to use BMTs as a tool to explore the flexibility of the onset of premigratory fueling and its rate given changes in foraging duration. We compared our baseline BMTs with “maximum foraging” BMTs, where geese exploited all available foraging time during daytime and nighttime. Geese started to increase foraging duration in late February, but body mass in baseline BMTs started to increase from late March onward due to a later decrease in energy expenditure and an increase in food quality. In maximum foraging BMTs, we found that an increase in foraging duration led to a 2-day advance in the onset of fueling, and geese reached body masses equal to departure body mass in baseline BMTs 4 days earlier, thereby also shortening their potential fueling period. Our sensitivity analysis shows that this difference of 4 days is robust to changes in parameter values on energy intake and expenditure. Nocturnal foraging appears to facilitate at least some leeway for geese to advance the start of energy deposition as well as realize faster energy deposition, which would allow advancements in migration departure. This result shows that, indeed, time available for foraging forms an important constraint in the rate of fueling, as earlier shown in shorebirds and passerines (Kvist and Lindström 2000; 2003). However, given observed phenological mismatches of up to 17 days in the breeding grounds (Lameris et al. 2018), a 4-day advancement of departure timing may not be enough for barnacle geese to cope with a warming climate.

Moreover, it is the question of whether geese, or birds in general, would be flexible enough to realize advancements at the start of premigratory fueling and increase fueling rates. First of all, geese may be unable to continue nocturnal foraging during spring as they require a higher threshold of maintenance activities, including resting and preening. This is, however, unlikely given that Arctic breeding birds, such as waterfowl and shorebirds, can forage for large parts of the 24-h day in the Arctic summer (Schekkerman et al. 2003; Klaassen et al. 2010). Second, geese may forego nighttime foraging in spring as it is less profitable or more dangerous compared to daytime foraging. Barnacle geese are known to actively select high-quality forage patches, plants, and plant parts (Prop and Deerenberg 1991; Durant et al. 2003), for which they probably rely on visual cues. Therefore, whether nighttime foraging is equally profitable depends on whether geese can be as selective at night as during the day. Especially, during spring when forage plant quality peaks, daytime foraging may be much more profitable compared to nighttime foraging, especially in diverse and heterogeneous natural habitats. It may matter less during winter, when forage plants generally are low in quality and geese may be less selective, and in homogeneous pasture monocultures, where tillers are generally of the same, high quality (Fox and Abraham 2017; Pot et al. 2019). Nevertheless, even in pastures, there may exist differences in the quality of patches (Bos et al. 2005), which could render nighttime foraging less profitable. In addition, there may be costs associated with nighttime foraging as geese foraging at night are vulnerable to mammalian predators. Moonlit nights appear to increase predation risk for birds in general, but moonlight may also allow birds to detect predators (Prugh and Golden 2014). While, in winter, the benefits of nighttime foraging may exceed the costs of increased predation risk (Brown and Kotler 2004), this may not be the case in spring when geese have ample time during the day to satisfy their energetic requirements.

Third, geese may be physiologically limited to quickly adopt earlier and more rapid energy deposition. Higher intake rates during spring fueling go hand in hand with increasing size and efficiency of digestive systems in migratory birds (van Gils et al. 2003; 2008), suggesting that rapid energy deposition may also be limited by the size of digestive systems (van Gils et al. 2003). While physiological development of digestive systems may be rapid in response to diet switches (Dekinga et al. 2001; Charalambidou et al. 2005), it is slower in waterfowl when facing a relatively slow increase in food quality (Van Gils et al. 2008; Kleyheeg et al. 2018).

While we do not have data to explore whether the onset or rate of premigratory fueling has been subject to change in recent years, no advances in migration departure have been observed for barnacle geese (Tombre et al. 2008; Eichhorn et al. 2009; Lameris et al. 2018). Optimal conditions for reproduction in the Arctic breeding grounds are generally advancing (Lameris et al. 2018; Fjelldal et al. 2020), but large annual stochasticity in the onset of spring (Box et al. 2019; Lameris et al. 2019), as well as contrasting effects of earlier springs on early and late stages of reproduction (Nolet et al. 2020), may not yet drive geese to advance their migratory departure from the wintering grounds. Nevertheless, in contrast to barnacle geese, which currently use few temperate staging sites and almost make a “jump” migration to the Arctic coast (Eichhorn et al. 2009), larger species that use a chain of temperate and Arctic stopover sites have in recent decades advanced their migration departure (Nuijten et al. 2020). Possibly, the inability of birds such as barnacle geese to predict conditions on distant staging sites hampers adjustments in the timing of energy deposition and migratory departure (Kölzsch et al. 2015).

## CONCLUSIONS

We find that barnacle geese balance their energy budget in winter by expanding foraging to moonlit nights in cold conditions. While we show that some leeway exists to advance and increase premigratory fueling by extending nighttime foraging in spring, this would not make up for the currently observed advancements in the optimal timing of reproduction at the breeding grounds. Moreover, the question remains if birds are not constrained to make use of this additionally available time, also as nighttime foraging may be less profitable compared to daytime foraging. Since birds require sufficient body stores in order to depart for migration, changes in migration timing are likely to be strongly constrained by the rate and onset of fueling prior to migration. Time constraints as well as inflexible timing of fueling can thereby limit the potential for migratory birds to adjust migrating timing in synchrony with climate warming in their breeding grounds.

## Supplementary Material

araa152_suppl_Supplementary_Information

## Data accessibility:

Analyses reported in this article can be reproduced using the data provided by Lameris et al. (2020).
