# Supplementary material for: Nocturnal foraging lifts time constraints in winter for migratory geese but hardly speeds up fueling
Source: Behav Ecol. 2021 Mar 25;32(3):539–52. doi: 10.1093/beheco/araa152 (PMC8177807; doi:10.1093/beheco/araa152)
Supplement: araa152_suppl_Supplementary_Information [file araa152_suppl_supplementary_information.docx]

**Supplementary material to “Night-time foraging lifts time-constraints in winter for migratory geese but fueling advancement is limited”**

**S1. Calculating flight costs**

For each GPS-fix *i*, we determined the great circle distance in m (*D_i_*) between this GPS fix and the following GPS fix (*i* + 1). To determine when the bird had flown in between GPS fixes, we calculated the minimum groundspeed *Vg min* (km/h) between these fixes as:

${Vgmin}_{i}= \frac{D_{i}}{t_{i}}$ (1)

If *V_g min_* > 0.28 m/s (= 1 km/h), we considered that the bird had flown the distance between the two GPS-fixes (Pot et al. 2019). For these points we then calculated the total time of flight between fixes (*t_flight_*) and the airspeed (*V_a_*) of the bird:

${tflight}_{i}= \frac{D_{i}}{{Vg}_{i}}$ (2)

${Va}_{i}= \sqrt{{Vg}_{i}^{2}+{Vw}_{i}^{2}-2*{Vg}_{i}*{Vw}_{i}*cos(\gamma-\omega)}$ (3)

where Vg_i_ is the ground speed, Vw_i_ is the wind speed, γ is the bearing of bird, ω is the wind direction. For Vg_i_ we use the average groundspeed for all flight events for all individuals, which equals 14.68 m/s. As for every GPS-fix we now have total duration and the time spent in flight, we correct the total duration *t_i_* for the time spent in flight by subtracting *tflight_i_* from *t_i._*. The energetic costs for the remaining time period (*t_i_* - *tflight_i_*) are calculated according to formula 1 (for behaviours grazing, active or inactive).

From the airspeed Va_i_, body mass W_i-1_ and basal metabolic rate BMR_i_ of the bird, we calculated the potential flight costs in kJ min^-1^ *PF* using the flight power theory developed by Pennycuick (Pennycuick 1989). We then calculated the actual flight costs *F_i_*:

$F_{i}={PF}_{i}*{tflight}_{i}$ (4)

**S2. Body mass trajectories model**

*2.1 Metabolisable energy intake*

*2.1.1 Study site*

We calculated metabolisable energy intake from data on food quality and foraging intake rates, by measurements on forage plants, goose droppings and dropping rates, collected in the wintering regions of migratory and resident geese. We conducted measurements on two representative wintering site for migratory geese: 1) the island of Schiermonnikoog in the Wadden Sea region (Eichhorn et al. 2012), and 2) the Dutch Delta region in the South-Western part of the Netherlands (van der Jeugd et al. 2009), where many resident and migratory barnacle goose spend the winter. We conducted measurements on Schiermonnikoog from early March – mid-May 2016 in agricultural fields (n = 3 plots) and salt marshes (n = 3), and measurements in the Delta from mid-February to mid-April 2017 in agricultural fields (n = 3) and natural grasslands (n = 3).

*2.1.2 Field and lab methods*

During measurements we collected fresh droppings and the upper leaves of the main forage plants for barnacle geese on Schiermonnikoog (van der Graaf 2006; van der Graaf et al. 2006): *Festuca rubra* on Schiermonnikoog saltmarshes, *Poa annua* in natural grasslands in the Delta, and *Lolium perenne* on agricultural fields at Schiermonnikoog and the Delta. Forage plants were collected in temporal exclosures every 1 – 2 weeks, fresh droppings were collected in these same fields, after having observed defecating geese which had been present for at least 2 h. We dried collected droppings and vegetation for 24 h at 60˚C. We weighed individual droppings to the nearest mg (see dropping mass, Fig S3) after which samples were grinded. We measured nitrogen concentration using a C:N analyser (Flash EA 1112 analyzer from Thermo Fisher Scientific Inc. Waltham, USA). We measured energetic value of samples (~ 0.5 g of dry material) using an IKA C5000 oxygen bomb calorimeter (see for energetic value of forage plants Fig S3). We chemically determined the mass concentration of an indigestible marker (acid detergent fiber content, ADF, following Prop and Deerenberg 1991) using ANKOM Technology (Macedon, NY, USA) for a subset of samples. In order to comprise a model to estimate ADF from near-infrared (NIR) measurements, we took NIR measurements from all samples using a NIR spectrometer (Bruker MPA FT-NIR analyser with OPUS 7.0.129 software). From the chemical ADF measurements, and a larger set of samples (food plants and droppings from Barnacle Geese collected at Kanin peninsula (RU, 68°24’N, 44°99’E) in 2002, Kolokolkova Bay in 2003 and 2015, Schiermonnikoog in 2004 and 2014–2016, Westplaat Buitengronden (NL, 51°79’N, 4°13’E) in 2015–2016, Eichhorn & Lameris, unpubl. data) we could successfully comprise a model which we used to estimate ADF for all samples. We determined the fraction of inorganic matter, and all chemical measurements were corrected to ash free dry matter basis (AFDM). We recorded the time between subsequent defecations of foraging geese (dropping rate *r*) in March – mid-May 2016 on Schiermonnikoog. We corrected for potentially missed observations in our dropping intervals using the package intRvals in R (Dokter et al. 2017). Dropping rates in salt marshes and pastures did not significantly differ from each other (t-test, t = -1.107, df = 31.8, p = 0.276). We therefore used a single dropping rate for all analyses, namely 289.2 ± 34.6 s.

*2.1.3 Calculating metabolisable energy intake*

Using sets of samples of forage plants and droppings which were collected at two week intervals we calculated the digestibility (Fig S3):

$D=1-\frac{{ADF}_{g}}{{ADF}_{d}}$ (5)

where ADF_d_ and ADF_g_ is the acid detergent fiber content of droppings and grass respectively. Thereafter, we calculated the metabolizable energy intake *I*, by first determining the rate of excretory energy output (*R_out_*_)_ and ingested energy input (*R_in_*) as:

$R_{out}=r*m_{d}*u_{d}$ (6)

$R_{in}= R_{out}*\frac{{ADF}_{d}}{{ADF}_{g}}*\frac{u_{g}}{u_{d}}$ (7)

where *r* is the dropping rate in droppings per minute, *m_d_* the dropping weight, *u_d_* and *u_g_* the energetic content of droppings and grass respectively. The rate of metabolised energy intake *I* (kJ / min foraging) can then be calculated as:

$I= R_{in}- R_{out}=q*R_{in}= \frac{q}{\left( 1-q \right)}* R_{out}$ (8)

where *q* is the apparent metabolisability, which equals:

$q=1- \frac{{ADF}_{g}}{{ADF}_{d}}*\frac{u_{d}}{u_{g}}$ (9)

As the relationship between *I* and *GDD* differed between habitats (interaction GDD and habitat: F_1,37_ = 4.29, p = 0.045), we then analysed the relationship between *I* and GDD for both agricultural and salt marsh habitats (Fig. S1, agricultural: 0.003 ± 0.001 increase in *I* per degree; salt marsh: 0.002 ± 0.001 increase in *I* per degree). From these linear regressions we predicted potential metabolisable energy intake rate *PI_i_* for our GPS-fix data from GDD, separately for geese grazing in agricultural pastures and salt marshes (Fig. S3d). We had little data for GDD < 300, we assumed a fixed *I* for these days, predicted from the linear regressions (for GDD = 300). We attained GDD (with a base temperature of 0°C) for every day in the dataset of our tagged birds, using temperature data from a range of weather stations in the Wadden Sea (2015) (Lauwersoog, data from www.knmi.nl; Wangerland, Sankt-Peter and List, data from www.dwd.de).

The metabolisable energy intake (kJ/min) was then calculated as:

$I_{i}= {PI}_{i}*t_{i}$ (10)

where *t_i_*  is the time interval from the GPS fix *i* until the following GPS fix *i* + 1

*2.2 Starting body mass*

As we did not weigh the individual birds during staging at the wintering grounds, we derived a correlation between body size (measured as tarsus length * head length) and body mass from measurements of 29 female geese caught during winter 2008 – 2009 in the province of Friesland, the Netherlands (Ens et al. 2008). Using this correlation (body weight = 0.2186*(tarsus length * head length) + 254.43, R^2^ = 0.52) we calculated body mass from tarsus and head length (as measured in summer 2014 for the individual migratory geese).

*2.3 Comparison body mass measurements*

We assessed our model fit by comparing body mass (*Wi*) of the migratory population with body mass measurements taken from 484 adult female barnacle geese over the season: 202 geese caught in several places in the Netherlands in January – February 2000 – 2009 (Müskens, unpublished); 29 geese caught in the Lauwersmeer region in January 2008 and 2009 (Ens et al. 2008); 77 geese caught in Schleswig-Holstein in March 1979 and 1989 (Ebbinge et al. unpublished); 67 geese caught in March 2016 and 2017 near Freiburg (Müskens, unpublished) and 109 geese caught at Schiermonnikoog in April 2004 (Eichhorn et al. 2012).


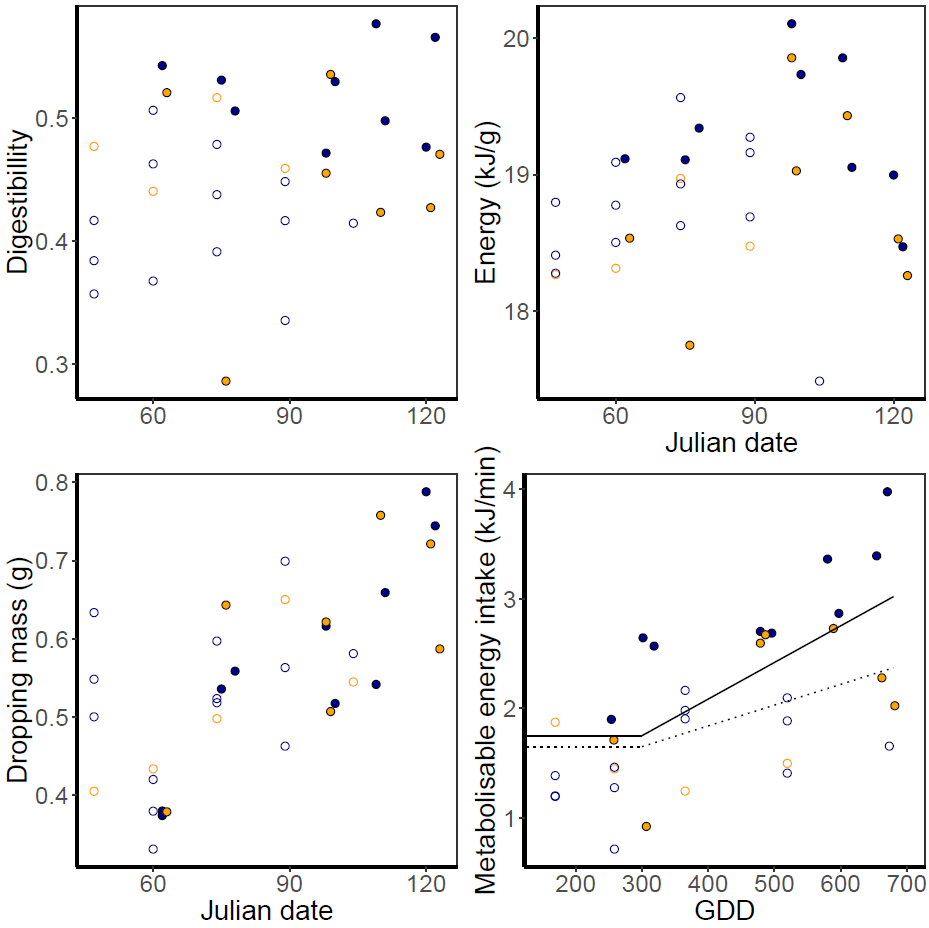


**Figure S1**: Digestibility and combustion energy content (ADFM corrected) in forage plants of barnacle geese and their dropping mass over day-of-the-year, and metabolisable energy intake over growing degree days (GDD) on wintering and spring staging sites on natural grasslands (orange) and agricultural grasslands (blue). Closed dots show data from Schiermonnikoog, open dots show data from the Delta. Lines show linear regressions from GDD=300 onwards (see methods), with a dotted line for natural grasslands and a solid line for agricultural grasslands.

**
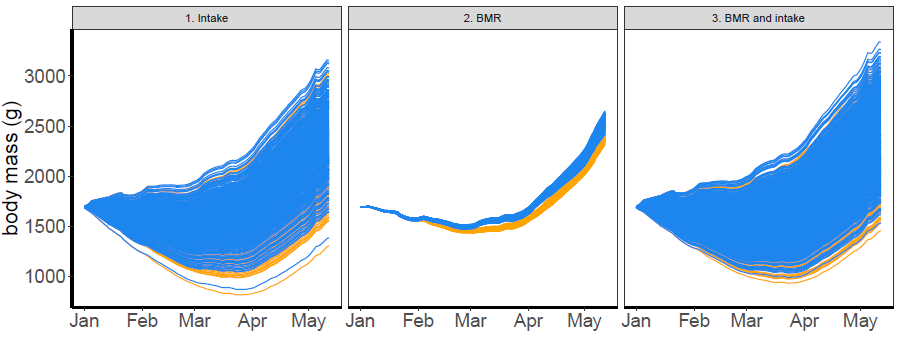
Figure S2**: The population average baseline BMT (orange) and ‘maximum foraging’ BMT (blue) per simulation in the sensitivity analysis. The three panels show simulation runs where new parameter variables were included for (1) only potential intake rate *PI*, (2) only basal metabolic rate *BMR_ref_* and (3) both parameters.

**Table S1**: Linear mixed effect models for daytime grazing (DG), night-time grazing (NG) and total grazing duration (TG) during the months January and February. Models include fixed effects day length (DL), day-of-the-year (DY), number of moonlit hours (MT) and thermoregulation costs (T), and individual goose as random intercept. Models are ordered from lowest to highest AICc values, with the best performing model marked **bold**.

|  | **model** | **degrees of freedom** | **AICc** | **delta AICc** | **model weight** |
| --- | --- | --- | --- | --- | --- |
| ***A) Daytime foraging duration (DG)*** | | | | | |
| **1** | **DG ~ DL** | 7 | 14325.4 | 0 | 0.951 |
| **2** | DG ~ DL + T | 8 | 14331.4 | 5.95 | 0.049 |
| **3** | DG ~ 1 | 6 | 14484 | 158.54 | 0 |
| **4** | DG ~ T | 7 | 14490.1 | 164.64 | 0 |
|  |  |  |  |  |  |
| ***B) Night-time foraging duration (NG)*** | | | | | |
| **1** | **NG ~ DY + MT + T** | 8 | 14212.3 | 0 | 0.968 |
| **2** | NG ~ DY + MT | 7 | 14219.2 | 6.83 | 0.032 |
| **3** | NG ~ DY + T | 7 | 14249.6 | 37.23 | 0 |
| **4** | NG ~ DY | 6 | 14258.3 | 45.95 | 0 |
| **5** | NG ~ MT + T | 7 | 14281.5 | 69.17 | 0 |
| **6** | NG ~ MT | 6 | 14288.1 | 75.72 | 0 |
| **7** | NG ~ T | 6 | 14335.2 | 122.88 | 0 |
| **8** | NG ~ 1 | 5 | 14343.3 | 131 | 0 |
|  |  |  |  |  |  |
| ***C) Total foraging duration (TG)*** | | | | | |
| **1** | **TG ~ DL + MT + T** | 9 | 15302 | 0 | 0.799 |
| **2** | TG ~ MT + T | 8 | 15305.8 | 3.73 | 0.123 |
| **3** | TG ~ DL + MT | 8 | 15307 | 4.97 | 0.066 |
| **4** | TG ~ MT | 7 | 15310.6 | 8.56 | 0.011 |
| **5** | TG ~ DL + T | 8 | 15340.3 | 38.27 | 0 |
| **6** | TG ~ T | 7 | 15344 | 41.93 | 0 |
| **7** | TG ~ DL | 7 | 15347.1 | 45.05 | 0 |
| **8** | TG ~ 1 | 6 | 15351 | 48.97 | 0 |

**Table S2:** Linear mixed effect models of body mass as calculated from body mass trajectories in the baseline BMT scenario and the ‘no night-time foraging’ scenario. Models include fixed effects day-of-the-year (DY) and BMT scenario (S), their interaction (DYxS), and individual goose as random intercept. Models are ordered from lowest to highest AICc values, with the best performing model marked **bold**.

|  | **model** | **degrees of freedom** | **AICc** | **delta AICc** | **model weight** |
| --- | --- | --- | --- | --- | --- |
| **1** | **BM ~ DY**x**S** | 5 | 20977.6 | 0 | 0.995 |
| **2** | BM ~ S | 5 | 20988.4 | 10.77 | 0.005 |
| **3** | BM ~ DY + S | 4 | 22411.4 | 1433.73 | 0 |
| **4** | BM ~ S | 4 | 24428.4 | 3450.79 | 0 |
| **5** | BM ~ 1 | 3 | 24769.8 | 3792.14 | 0 |

**References**

Dokter AM, van Loon EE, Fokkema W, Lameris TK, Nolet BA, van der Jeugd HP. 2017. Analyzing time-ordered event data with missed observations. Ecol Evol. 7(18):7362–7369.

Eichhorn G, Meijer H, Oosterbeek K, Klaassen M. 2012. Does agricultural food provide a good alternative to a natural diet for body store deposition in geese? Ecosphere. 3(April):1–13.

Ens BJ, Barlein F, Camphuysen KCJ, de Boer P, Exo K-M, Gallego N, Hoye B, Klaassen R, Oosterbeek K, Shamoun-Baranes J, et al. 2008. Tracking of individual birds. Report on WP 3230 (bird tracking sensor characterization) and WP 4130 (sensor adaptation and calibration for bird tracking system) of the FlySafe basic activities project. Beek-Ubbergen.

van der Graaf AJ. 2006. Geese on a green wave : Flexible migrants in a changing world. Rijksuniversiteit Groningen.

van der Graaf AJ, Stahl J, Klimkowska A, Bakker JP, Drent RH. 2006. Surfing on a green wave-how plant growth drives spring migration in the Barnacle Goose Branta leucopsis. Ardea. 94(3):567–577.

Lameris TK, Jochems F, van der Graaf AJ, Andersson M, Limpens J, Nolet BA. 2017. Forage plants of an Arctic-nesting herbivore show larger warming response in breeding than wintering grounds, potentially disrupting migration phenology. Ecol Evol. 7(8):2652–2660.

Pennycuick CJ. 1989. Bird flight performance. Oxford, United Kingdom: Oxford University Press.

Pot, M.T., de Koning, S., Westerduin, C., de Boer, W.F., Shariati, M., Lameris, T.K. 2019. Wintering geese trade-off energy gain and costs when switching from agricultural to natural habitats. Ardea 107 (2): 1 – 14.

Prop J, Deerenberg C. 1991. Spring staging in Brent Geese Branta bernicla: feeding constraints and the impact of diet on the accumulation of body reserves. Oecologia. 87(1):19–28. doi:10.1007/BF00323775.
